# Psychological Intervention for Improving Cognitive Function in Cancer Survivors: A Literature Review and Randomized Controlled Trial

**DOI:** 10.3389/fonc.2015.00072

**Published:** 2015-03-25

**Authors:** Summer King, Heather Joy Green

**Affiliations:** ^1^Menzies Health Institute Queensland and School of Applied Psychology, Griffith University, Gold Coast, QLD, Australia

**Keywords:** cancer, cognitive function, cognitive rehabilitation, group, randomized controlled trial, survivorship

## Abstract

Although the impact of cancer and associated treatments on cognitive functioning is becoming an increasingly recognized problem, there are few published studies that have investigated psychological interventions to address this issue. A waitlist randomized controlled trial methodology was used to assess the efficacy of a group cognitive rehabilitation intervention (“ReCog”) that successfully targeted cancer-related cognitive decline in previously published pilot research. Participants were 29 cancer survivors who were randomly allocated to either the intervention group or a waitlist group who received the intervention at a later date, and 16 demographically matched community volunteers with no history of cancer (trial registration ACTRN12615000009516, available at http://www.ANZCTR.org.au/ACTRN12615000009516.aspx). The study was the first to include an adapted version of the Traumatic Brain Injury Self-Efficacy Scale to assess cognitive self-efficacy (CSE) in people who have experienced cancer. Results revealed participating in the intervention was associated with significantly faster performance on one objective cognitive task that measures processing speed and visual scanning. Significantly larger improvements for the intervention group were also found on measures of perceived cognitive impairments and CSE. There was some evidence to support the roles of CSE and illness perceptions as potential mechanisms of change for the intervention. Overall, the study provided additional evidence of feasibility and efficacy of group psychological intervention for targeting cancer-related cognitive decline.

## Introduction

Research supports a relationship between cancer and associated treatment and subsequent cognitive impairment in some cancer survivors (1–3). Reported changes in areas like memory, attention, executive function, and processing speed have been linked to type of cancer and advancement of the disease (4), treatments (5), as well as other issues like increased stress and fatigue (6). As cognitive impairment has the potential to impact quality of life (QoL), relationships, and adjustment to occupational functioning after cancer (7), it is an important survivorship issue.

Although the impact of cancer and associated treatments on cognitive functioning is increasingly recognized, relatively few published studies have investigated psychological intervention programs to address this issue in survivors of adult-onset cancers. The theoretical basis for such interventions and results of previous studies will be discussed before describing new randomized controlled trial (RCT) findings.

## Background

### Cancer and cognition

Reviews assessing the impact of cancer treatments on cognition, in the absence of known primary or secondary tumors in the central nervous system (CNS), have indicated cognitive dysfunction frequencies ranging from 13 to 75% (8–10). A recent meta-analysis that examined 13 studies including a range of cancer types and cognitive domains found executive function to be most affected by chemotherapy and found evidence for impairment in language and memory (11). Seventeen studies met inclusion criteria for another meta-analysis and findings indicated significant cognitive deficits to be limited to verbal and visuospatial abilities (12). Despite variability in these domains, the meta-analyses have demonstrated a consistent relationship between cancer and cognitive impairment. Dysfunction following treatment is often considered an acute issue that should subside within months after treatment (13) and some research has indicated no long-term differences in cognitive function between people with and without a history of cancer (14). However, longitudinal research has also shown that cognitive complaints following treatment may endure for up to 10 years (15). Several studies have highlighted the impact of type of cancer and treatments including influence of treatment received (16–19). It is therefore suggested that a range of factors may contribute to the variability in prevalence of cognitive dysfunction after cancer.

#### Objective and subjective cognitive function

Cognitive dysfunction is measured using both objective and subjective forms of assessment. Objective measures often provide information about specific areas of cognition (e.g., working memory) allowing for comparison of different domains. Prevalence rates of dysfunction are primarily based on objective measures, but subjective cognitive impairment is reportedly more prevalent in cancer survivors (9). Subjective measures assess self-perceptions of cognition including cognitive functioning in a person’s daily life, and involve self-report assessments such as questionnaires (9). One large-scale study assessed 1933 breast cancer survivors, compared to 500 healthy controls, on a range of subjective measures of cognition and found statistically significant differences between groups (9% of survivors reported subjective cognition in a problematic range vs. 2.8% for controls) (20).

The association between objective and subjective cognitive impairment in people with cancer is inconsistent, with some studies finding a positive association but a higher number of studies finding no relation between these different forms of measurement (8, 9). An explanation for the discrepancy between objective and subjective measures of cognitive function is that subjective impairment is often more an indicator of psychological distress than a measurement of cognition (8, 21), and several studies have indicated a relationship between perceived cognition and psychological factors like mood and distress. It is therefore suggested that subjective and objective measures of cognition should be treated as separate assessments and that those who self-report cognitive concerns should also be screened for other psychological concerns (17).

Other proposed issues include the potential poor ability of objective measures to assess the subtle cognitive changes that may occur as a result of cancer treatment (22) and the need for a more comprehensive evaluation of mood so the influence of psychological factors can be accounted completely. In light of the variable evidence regarding cancer-related cognitive decline and complex relationships impacting cognition (e.g., objective and subjective function, psychosocial factors), it is helpful to consider conceptual models designed to assist with interpretation of the research findings.

#### Explanatory models

Models have been proposed in order to summarize the relationships among physiological and psychological factors associated with cancer and cognitive impairment (23–25). Hess and colleagues included 70 articles in a systematic review, and developed a conceptual model of pathways by which cancer treatments may lead to changes in cognitive functioning (24). Their model incorporates antecedents; mediating factors and associated toxicities; moderators; and consequences for treatment-related cognitive dysfunction. The model illustrates that multiple factors impact on subjective/objective cognitive function (grouped together), and consequently on QoL and functional ability. The model also accounts for moderating factors which have been shown to influence the relationship between cancer and cognition (24). When considering how specific cancer types (termed an “antecedent” in this model) may be associated with changes in cognitive function, one issue of note is that various adult-onset cancers have different rates of CNS metastases. The frequency of metastases may be underreported because specific investigations for such metastases may not occur unless symptoms of CNS dysfunction are evident. Another point of note is that the model lists varied toxicities that potentially link some cancer treatments to cognitive changes for some patients, including anemia, cytokines, hormonal status, and vascular injury, as well as direct neurotoxicity. Therefore, the evidence does not support one specific, universal mechanism for all cancer-related cognitive dysfunction.

A model by a different research group proposed predictors of both subjective and objective cognitive function in people with cancer (23). The model suggests cancer treatments, emotional health, and physical health to be predictors of objective cognitive impairment and that emotional health and objective impairment may predict subjective cognitive impairment. These authors noted that, in many instances, subjective measures of cognition are more strongly related to psychosocial factors such as coping, emotions, and personal interpretations of a situation (termed “appraisal”), than to objective cognitive function. Furthermore, their model suggests psychosocial elements including appraisal and coping can impact the level of emotional distress and consequently correlate with physical health. It is proposed that the model may inform interventions by incorporating assessment of individual vulnerabilities and current difficulties, assisting patient education regarding current and prospective cognitive function, and identifying potential areas for remediation (23).

Vearncombe and Pachana reviewed 22 studies to evaluate the impact of treatments, health, and psychological factors on cognition for women with breast cancer (26). They proposed that indirect factors, including psychological well-being, may influence cognitive performance and found a major gap in the literature in terms of study of the impact of these indirect factors on cognition after cancer treatment. Their research highlighted the potential contribution of psychological variables to cognitive performance for cancer survivors, including the potential to intervene with psychological approaches even when biological causes may contribute.

### Psychosocial variables

#### Quality of life

A psychological variable that has been researched in terms of its relationship to cancer and cognitive function is QoL. QoL has been described as subjective perception of how well a person functions across areas of their life and is domain-specific encompassing the interactions of psychological, physical, social, and spiritual well-being (27). Areas of QoL impacted by cancer are commonly reported to include areas like physical, sexual, role, and social functioning (28). Meta-analyses in this area have even supported use of QoL as a prognostic indicator of survival in some people with cancer (29, 30).

A systematic review of 28 studies showed worse outcomes on QoL are reported significantly more frequently by women with breast cancer than community controls (31). QoL impacted by breast cancer was related to reduced physical functioning, premature menopause, and the impact of psychosocial outcomes as a result of diagnosis and treatment (e.g., depression). Another review examining the long-term impact of cancer on QoL indicated that survivors (at least 5 years post-treatment) reported good overall QoL but reported issues with specific areas of QoL like sexual functioning (32). Predictors of better QoL included fewer current medical issues, better social support, and higher income; chemotherapy treatment was a predictor of worse scores on measures of QoL.

Although limited, there is some research into the impact of cancer-related cognitive decline on QoL. Research has shown perceived cognitive impairment after cancer treatment to be linked to reduced QoL, daily functioning, reduced work efficiency, and negative reactions from others (7). One study of health-related QoL in 76 people with cancer found individuals with higher subjective cognitive deficits reported worse health-related QoL (33). A qualitative study of men randomized to androgen-suppressing medication for prostate cancer found that decreased cognitive function was the most frequent change in behavior or symptoms that participants attributed to their medication (34). Overall, existing research indicates that there is a need for more research investigating the relationship between cognitive functioning and QoL in cancer survivors.

#### Psychological distress

Research supports that diagnosis of cancer and treatment may lead to increases in mental health issues including depression and anxiety (35–37). A meta-analysis of 58 studies that investigated psychological outcomes related to cancer diagnosis found varied results in terms of significantly less psychological problems when compared to a psychiatric population, but significantly higher levels of depression than a “normal” population (38). Among a sample of 1083 people with breast cancer, at least 40% had one psychological diagnosis, 38% exhibited moderate-high rates of anxiety, 22% reported moderate-high depression, 12% exhibited post-traumatic stress disorder, and 7.8% met diagnostic criteria for all three diagnoses (39). Increased rates of comorbid depression have also been correlated with the advancement of the disease (40).

It has been reported that psychological disorders like depression and anxiety may impact cognitive function in the general population and a range of clinical populations (41, 42). Depression has been particularly linked to deficits in attention and a large study that examined depression, anxiety, and cognitive function in an elderly population found a significant and almost linear relationship between depression and objective measures of impaired cognitive function (41). Some research has not found a correlation between psychological distress and objective cognitive function in people with cancer (13, 43, 44). However, a number of studies have shown a relationship between mental health and subjective cognitive impairment in this population (13, 18). These findings indicate that subjective complaints regarding cognitive impairment may be more revealing of emotional distress than objective cognitive impairment (18), and also suggest there may be other psychosocial variables that impact psychological well-being in this population.

#### Fatigue

Survivors of cancer often report persistent fatigue and it has been found to affect individuals irrespective of type of cancer or treatment received (45, 46). Cancer-related fatigue has been associated with QoL (47) and psychosocial well-being (35). A systematic review including 44 studies demonstrated consistently more fatigue in cancer groups than the general population with prevalence ranging between 39 and 90% (48). Research also suggests up to a third of people treated with radiation or chemotherapy continued to experience fatigue 5–10 years after treatment was completed (49, 50). Cancer-related fatigue may also be associated with financial burden as it can impair an individual’s ability to work and perform activities of daily living (51).

The relationship between fatigue and cognitive functioning is well established in a range of clinical populations including multiple sclerosis (52) and chronic fatigue syndrome (53), but results have been mixed when investigating populations with cancer. One study found no differences between severely fatigued cancer survivors, non-severely fatigued cancer survivors, and the control group on objective neuropsychological assessments; but the severely fatigued group scored significantly worse on self-report assessments of cognitive functioning (54). These results suggested that cancer-related fatigue may be associated with subjective but not objective cognitive functioning. This is supported by other studies, which have failed to find a relationship between fatigue and objective cognitive dysfunction in cancer survivors (55, 56).

#### Benefit finding

Benefit finding refers to the potential for individuals who have experienced cancer or other potentially traumatic events to view aspects or outcomes of their experience as positive or beneficial (57). Positive experiences post-diagnosis for cancer as reported by some people may include increased sense of spirituality and purpose, improved relationships, and increased skills (58).

A meta-analysis of 87 studies investigating benefit finding in populations with cancer found the construct was related to measures of positive well-being and less depression but was not related to measures of anxiety or global distress (59). It was suggested from this research that benefit finding may be important to consider when researching survivorship issues as it appears to represent positive outcomes from illness as opposed to “a mere lack of distress” (59).

#### Cognitive self-efficacy

The relationship between confidence in ability to perform cognitive tasks and objective measures of cognitive performance is a robust finding in the literature (60, 61). Cognitive self-efficacy (CSE) refers to an individual’s confidence and/or perceptions regarding the effectiveness of their cognitive functioning in expected situations (62). An individual with low CSE may avoid tasks they believe to exceed their abilities, for example, they may not feel that they can solve problems related to their cognitive complaints. In contrast, another person with high CSE may attempt more challenging tasks, viewing them as goals rather than threats (63).

Research suggests that among individuals with physical disease or disablement, functional disability is more strongly predicted by perceived self-efficacy than by the level of impairment or duration of illness (64, 65). Specific to cognitive dysfunction, studies have found CSE and cognitive complaints to be more closely related than CSE and cognitive capacity (66–68). Measures of self-efficacy have also been shown to predict cognitive performance independently of the individual’s level of skill (69, 70).

Several studies have measured aspects of CSE across cognitive training programs (64, 71). Results have generally found that as CSE increases, cognitive performance improves, and where self-efficacy has not increased, there have also not been substantial gains in objective performance (71). The theory that CSE may mediate the degree of improvement during cognitive training programs, such that an increase in CSE facilitates a more positive outcome, has also been suggested (64). Further research into the role of CSE in cognitive rehabilitation programs is therefore warranted as it “may have considerable heuristic and explanatory value for understanding the effective ingredients of interventions” (64) (p. 949).

#### Illness perceptions

Illness perception is a construct encompassing an individual’s appraisals and beliefs about their illness (72). A self-regulation framework posits that an individual’s perceptions of their illness lead to their choices of coping strategies for dealing with an illness (73). A meta-analytic review of 45 studies found people who perceive their illness as highly symptomatic use avoidance coping strategies in contrast to people who view their illness as curable/controllable and show more positive social functioning, improved mental health, and reduced distress and disease states (74).

Preliminary evidence from one psychological intervention study for cognitive impairment in cancer survivors showed a significant improvement for intervention participants on one subdomain of illness perceptions (75). This was “illness coherence” regarding cognitive problems, which refers to an individual’s beliefs about how well they understand the health problem. It has been suggested that if successfully targeted by interventions, improvement in illness perceptions would likely be related to improvement in a range of illness and psychosocial outcomes (76). Thus, the illness perceptions construct shows promise as a mediator of outcomes in psychosocial intervention studies but there has been little research to test this in the context of cognitive concerns for cancer survivors.

### Interventions for cancer-related cognitive dysfunction

#### Pharmacological interventions

A number of pharmacological agents have been trialed for their use in addressing cognitive impairment after cancer. Reviews have identified erythropoietin, methylphenidate, and modafinil as pharmacological agents that may reduce cognitive impairment following treatment (25, 77). Despite these promising results, there are studies which have not shown any improvement in cognitive performance with medications (78) and it is noted that pharmacological treatments often have side-effects (79). For example, the presence of erythropoietin receptors in many cancers may raise concerns about potential increased risk of tumor growth or recurrence with use of erythropoietin to address these issues. Therefore, consideration of non-pharmacological, psychosocial interventions is important.

#### Psychosocial interventions

Published research has only relatively recently reported psychological interventions for cognitive dysfunction following treatment for adult-onset non-CNS malignancies. Many of these studies were published after the current study began in 2012. Some studies have focused on cognitive training, such as computerized exercises designed to strengthen relevant cognitive processes. Other studies have taken a broader cognitive rehabilitation approach, which may include cognitive skills training but also incorporates program elements such as psychoeducation, compensatory strategy training, and between session homework tasks (80). Cognitive rehabilitation usually incorporates cognitive behavioral therapy (CBT) principles. Some studies have used individually delivered interventions, whereas others have taken place in a group format. Please see Table 1 for information on psychological interventions addressing cancer-related cognitive impairment.

**Table 1:** **Psychological intervention studies addressing cognitive impairment in cancer survivors**.

Reference	Design	Participants	Intervention	Results
(81)	RCT (intervention vs. waitlist)	78 adults aged 65+ years with a history of chronic disease (*n* = 11 history of cancer)	Cognitive Behavioral Model of Everyday Memory (CBMEM): efficacy and awareness building, health promotion, strategy use, and relaxation. Group intervention, 8 sessions of 1.25 h each, over 4 weeks	Cancer survivors in intervention group improved in short-term memory on the Rivermead Behavioral Memory Test, memory self-efficacy, and metamemory. (Note: not planned as a cancer substudy)
(82)	Single-arm study	29 women at least 3 years post-chemotherapy for breast cancer	Memory and Attention Adaptation Training (MAAT): education, self-awareness training, self-regulation, compensatory strategies. Individual therapy, 4 sessions of 30–50 min each, once per month, plus up to 3 phone calls and participant workbook	Significant improvements in neuropsychological test performance, self-reported cognitive function, and QoL
(83)	Partially Randomized Controlled Trial (two interventions vs. treatment-as-usual)	96 women post-chemotherapy for stage I or II breast cancer, undergoing inpatient cancer rehabilitation	Neuropsychological Training Group: small group functional training and compensatory strategies for memory and attention in everyday situations. Computer intervention: individual therapist support for using software addressing memory/attention. Both groups attended 4 1-h sessions per week during their stay in hospital (3–5 weeks)	Improvements across most neuropsychological measures for all participant groups (i.e., no effects were specific to the interventions)
(84)	RCT (intervention vs. active control)	267 adults aged 65+ years; 22 cancer survivors: 14 intervention group, 8 control group	CBMEM (see above) compared to health information control condition	Cancer survivors in CBMEM declined less in visual memory performance over 14 months and improved more than control group on subjective memory measures. (Note: not planned as a cancer substudy)
(85)	RCT (two interventions vs. waitlist)	88 breast cancer survivors	Memory training: group memory exercises and skills practice. Processing speed: computerized training using increasingly difficult processing tasks. Both interventions used 10 1-h training sessions in small groups over 6–8 weeks	Both intervention groups improved neuropsychological test performance more than waitlist group, but processing speed training showed earlier benefits and generalized to memory performance whereas memory training not associated with changed processing speed. Both showed improvements in subjective cognition, QoL, and distress
(86)	RCT (intervention vs. waitlist)	40 women 18-months post-treatment for breast cancer	MAAT (see above)	Intervention group improved significantly more than waitlist participants on verbal memory (California Verbal Learning Test) and one QoL subscale (spiritual well-being)
(75)	Non-Randomized Controlled Trial (intervention vs. waitlist vs. community)	55 participants. 32 cancer survivors >4 months post-treatments; 23 community comparison	Responding to Cognitive Concerns (ReCog): education, compensatory and enhancement strategies for memory, attention, emotional adjustment, sleep, and fatigue. Group sessions lasting 2 h held weekly for 4 weeks, participant workbook/homework	Significantly greater improvement on overall cognitive function, immediate memory, visuospatial/constructional, and delayed memory measures for intervention group. Reduction in subjective cognitive impairment and distress for intervention group
(87)	RCT (intervention vs. waitlist)	28 adult cancer survivors >6 months post-treatment	Workshops addressing memory aids, memory skills, and mindfulness meditation. Group sessions lasting 1 h held weekly for 7 weeks	Intervention group improved significantly more than waitlist group on digit span and subjective cognition
(88)	Single-arm study	27 women 1.5–5 years post-treatments for breast cancer. *n* = 8 for EEG substudy	Cognitive rehabilitation, targeting attention, executive and memory challenges. Group sessions lasting 2 h held weekly for 5 weeks, participant workbook/homework	Significant improvements on Symbol Digit, Stroop reaction time, Trails A time, and subjective cognition. Increase in EEG alpha power was associated with improved subjective cognition at 2-month follow-up
(89)	RCT (intervention vs. waitlist)	41 breast cancer survivors	Online, computerized training program targeting executive function. Individual, home-based sessions lasting 20–30 min conducted 4 times per week for 12 weeks	Adherence was high. Intervention group improved significantly more on Wisconsin Card Sort Test, letter fluency, and symbol search, as well as some aspects of subjective executive function

The studies described in Table 1 show support for both group and individual psychosocial interventions for this clinical issue. In the limited research to date, no specific advantage has been shown for interventions with a broader cognitive rehabilitation approach compared to more focused cognitive training. Research has supported beneficial effects of psychological interventions for cancer-related cognitive impairment in studies of mixed tumor types (75, 81, 84, 87) as well as in studies limited to women treated for breast cancer (82, 83, 85, 86, 88, 89).

However, there are limitations in the research to date. Only one of the studies described in Table 1 included a matched attention control condition, and did demonstrate additional beneficial intervention effects in comparison to the health information arm (84). However, this research context was not specific to cancer survivors (84), so it is unclear whether an attention control that more specifically focuses on the concerns of cancer survivors might have additional benefits. The one study that did not find any additional benefit of psychological interventions designed to improve cognitive performance in comparison to treatment as usual was conducted in conjunction with inpatient cancer rehabilitation received by all participants, so it could be that the overall rehabilitation program acted as an attention control (83).

Psychological intervention studies to date have had relatively small sample sizes, and in some instances the findings associated with the intervention have represented only a small proportion of the statistical comparisons within the studies. Measures have varied, so it is difficult to make direct comparisons between studies. The majority of studies either investigating or intervening for cognitive impairment in cancer survivors with no known CNS tumors have been limited to samples with female breast cancer survivors and people treated with chemotherapy, making generalization of results to males and other cancer types difficult. Nevertheless, cancer-related cognitive impairment has been found in both sexes, in association with a range of cancer types and treatments, and clinically significant responses to cognitive rehabilitation have been found following colorectal, prostate, and testicular cancer as well as breast cancer (75). Moderators and mediators of intervention effectiveness are yet to be identified.

### Overview of current study

The present study aimed to test the efficacy and potential psychological mechanisms of a group intervention (ReCog) for cancer survivors targeting cognitive decline. Design elements intended to address gaps in the literature included incorporating a range of cancer types, using RCT design, and testing relevant psychological outcomes including potential explanatory variables.

It was hypothesized that there would be significantly greater improvements on objective cognitive function for participants in an intervention group than for participants in waitlist and community groups (Hypothesis 1). Similarly, it was predicted that the intervention group would show significantly greater improvements than other participants in subjective cognitive function (Hypothesis 2); in psychosocial measures including QoL, fatigue, distress, and benefit finding (Hypothesis 3); and in potential psychological explanatory variables of CSE and illness perceptions (Hypothesis 4). It was predicted that improvements in CSE and illness perception would be significantly associated with greater improvements in objective and subjective cognitive function (Hypothesis 5).

## Materials and Methods

### Participants

There were 16 intervention participants (aged 37–65, *M* = 50.4, SD = 8.8 years), 13 waitlist control participants (aged 40–72, *M* = 51.8, SD = 9.4), and 16 community comparison participants (aged 27–77 years, *M* = 52.9, SD = 4.3). Intervention and waitlist participants had experienced adult-onset cancer, excluding cancer affecting the CNS, and had completed major treatments for cancer at least 6 months prior. A further requirement was that these participants report subjective cognitive impairment on the EORTC Cognitive Functioning Subscale prior to the intervention (score of less than 100). Inclusion criteria for the community comparison group stipulated participants to be adults (over 18 years) who had never been diagnosed with cancer. RCT participants were recruited in 2012 and 2013 via Griffith University email and cancer support groups. Community comparison participants were recruited in 2009 and 2010 via contacts of the research assistants for a parallel psychometric study of 36 participants, from which 16 were selected to match the intervention participants for sex, age, and years of education. Statistical analyses revealed no significant differences among groups on demographic variables at baseline.

Cancer survivors were randomly allocated to intervention (*n* = 16) and waitlist control groups (*n* = 14). Randomization was conducted by a colleague unconnected to the research project who used a random number table to generate the allocation sequence and prepared numbered opaque envelopes that were opened at the end of the initial assessment session. One participant who was randomly allocated to the waitlist group was excluded from analyses due to a pre-existing neurological condition.

### Measures

#### Primary outcomes: cognitive measures

The Repeatable Battery for Assessment of Neuropsychological Status (RBANS) (90) is a 30-min battery that assesses objective cognitive function. Good validity and reliability of the RBANS have been reported (91, 92), with strong internal consistency in clinical populations, e.g., total score Cronbach’s alpha of 0.84 in people with traumatic brain injury (92).

The Trail Making Test (TMT) assesses attention, spatial organization, visual scanning, executive function, speed of processing, and mental flexibility (93). The TMT has exhibited good validity and reliability and is particularly sensitive to neurological impairment (93, 94).

The Functional Assessment of Cancer Therapy – Cognitive Scale Version 3 (FACT-Cog 3) (95) was designed for people with cancer and is used to measure areas of subjective cognitive function including perceived impairment, perceived ability, comments from other people regarding cognition, and QoL. Research has found high internal consistency for subscale scores on Version 2 of the scale, which has similar items and subscales to Version 3 (96).

The Brief Assessment of Prospective Memory (BAPM) (97) was used to assess subjective prospective memory. There are two subscales comprising the BAPM: the basic activities of daily living (BADL, e.g., forgetting to lock the door when leaving home) and instrumental activities of daily living (IADL, e.g., leaving the iron on). The BAPM has shown Cronbach’s alpha of 0.74–0.76 for subscales (97).

#### Secondary outcomes: psychosocial measures

The European Organization for Research and Treatment of Cancer Core Quality of Life Questionnaire (EORTC-QLQ-C30) (98) is designed to assess aspects of QoL relevant to cancer. Recent research demonstrates high internal reliability with Cronbach’s alpha above 0.80 for the functional scales (99), and reliability and consistency across cultures (100).

The Kessler Psychological Distress Scale (K10) (101) is designed to measure psychological distress including depression and anxiety. Research has indicated high levels of internal consistency, concurrent validity, and discriminant validity for the K10 (101).

The Benefit Finding Scale (102) assesses perceptions of positive contributions to life due to cancer diagnosis and treatment. Cronbach’s alpha of 0.90 or higher has been reported (103).

The current study adapted the Traumatic Brain Injury Self-Efficacy Scale (104) to assess CSE. Items ask participants to rate confidence that they can manage their symptoms related to their TBI or cognitive disorder. This wording was adapted slightly so that it referred to “symptoms related to your cancer-related cognitive difficulties.” The original scale was piloted with 21 military veterans experiencing mild cognitive disorder and with a history of TBI (104). Reliability of the adapted scale was assessed to be good for the current sample (Cronbach’s alpha = 0.91–0.95).

The Brief Illness Perception Questionnaire (B-IPQ) (72) was designed to assess cognitive and emotional representations of illness. Cancer survivor participants completed this measure in relation to “your cognitive difficulties” and community participants were asked to respond by imagining what they thought it would be like to experience “cognitive difficulties, such as a problem with your attention or memory.” Questions address issues like personal concern about cognitive difficulties, beliefs about benefit of treatment, and control over their difficulties. Previous research showed good test–retest reliability over 3- and 6-week time periods (*r* = 0.42–0.75) and good concurrent validity (72).

A participant satisfaction survey was completed by participants in the intervention group after the final group session (75).

### Intervention

The current study implemented an intervention that was previously developed and evaluated in an initial feasibility study (75). The intervention was titled “Responding to Cognitive Concerns (ReCog): a four session cognitive rehabilitation program for adults recovering from cancer.” The program comprised four topics: (1) aging, health, cancer, and cognitive function; (2) memory; (3) attention; and (4) fatigue, emotions, and cognition. The program is manualized for clinicians (105) and participants (106). The intervention included four 2-h sessions held weekly across 4 weeks and participants were required to complete homework between sessions. Each session included psychoeducation, group discussion, and skill development and application (75). The three intervention groups of three to eight participants were co-facilitated by two psychologists, offered at no cost, conducted at university campuses, and also offered to all waitlist control participants once they had completed assessments. Waitlist participants were able to seek any medical or health services they required during the study, with no restrictions apart from not being eligible to undertake the ReCog intervention until they had completed data collection.

### Procedure

The study was approved by the Griffith University Human Research Ethics Committee (PSY/16/12/HREC) and met the required regulatory standards for research with human participants. The trial was registered with the Australian New Zealand Clinical Trials Registry (ACTRN12615000009516). Data were collected at Griffith University in South-East Queensland, Australia. Baseline assessments took place within 2 weeks before the intervention commenced. At Time 1, participants completed objective measures of cognitive function followed by questionnaires, and group assignment was then revealed. At Time 2 and Time 3 assessments (within 2 weeks of intervention completion and at 3 months post-intervention), all participants were asked to complete the assessment battery.

Time 1 assessments were conducted by the first author, and Time 2 and 3 assessments were conducted by independent psychologists who were blind to the participant’s group membership. Participants in the community comparison group completed the same assessment battery as participants in the other two groups (excluding three of the questionnaires), across Times 1 and 2 only.

### Statistical analyses

Subscales and total scores for questionnaires were calculated using pro-rating methods suggested in the EORTC-QLQ-C30 scoring manual (107). The algorithms computed for each measure calculated the mean of all completed items where there was a minimum of 50% response and then substituted this value for the missing items.

Analysis of data from the RCT participants was conducted using 2 (Group) × 3 (Time) mixed factorial analyses of variance (ANOVA). Analysis of all three groups was conducted using 3 (Group) × 2 (Time) ANOVA. Simple effects analysis was used to follow up significant interactions. Planned contrasts comparing the intervention group to the waitlist group and to the community comparison group were used to follow up any effects of group. Effect sizes were calculated following guidelines for pre-test–post-test control group designs, using the Cohen’s *d* approach (108).

A sample size of 40 RCT participants was planned, based on *a priori* power analysis showing that this would yield more than 80% power for detecting Group × Time interactions for primary outcome measures, at α = 0.05 and with effect size estimated as Cohen’s *d* = 0.5–1.0 from previous research. Personnel and financial resources allowed 30 participants to be recruited for the RCT during the time available for the study, which was computed to provide adequate power based on previous estimates of effect. Intention-to-treat (ITT) analysis was conducted in the case of two participants who missed two assessments (109). There was no difference to the pattern of results when these two cases were included or excluded, and so these cases were excluded from relevant longitudinal analyses to provide “completer” analyses.

Clinical significance and reliable change scores were calculated with the clinical cut-off score being 1 SD below the Time 1 mean of the community comparison group. For measures where there was no data for this group (e.g., CSE), the Time 1 mean and SD for the waitlist group was used. The Reliable Change Index (RCI) was calculated using a formula, which includes a correction for practice effects as a result of test–retest designs (110). On the basis of clinical significance and reliable change scores, individuals were classified into change categories (recovered, improved, unchanged, deteriorated, or false positive) and the frequencies of the change categories were compared between groups using Fisher’s Exact Test.

To investigate potential mechanisms of change, change scores between Time 1 and Time 2 were calculated for CSE and illness perceptions. To be candidate mechanisms of effect, these variables would need to be associated with receiving the intervention and to show changes that preceded change on outcome measures (111). Therefore, the change scores for CSE and illness perceptions were investigated for correlations with group assignment (intervention or waitlist) and with objective and subjective cognitive changes between Time 1 and Time 3.

## Results

### Data screening and preliminary analyses

One intervention participant withdrew after Time 1 assessment and one waitlist participant did not complete Time 2 questionnaires or any Time 3 assessment. Missing data for specific questionnaires at single time points reduced the sample size slightly for analyses of Benefit Finding (by three intervention participants and two waitlist participants), K10 (one intervention and one waitlist participant), and FACT-Cog 3 (one waitlist and one community participant). There were no other missing data.

Several variables were skewed. Because transformations to correct skewness did not change the pattern of results, the untransformed data were retained for analyses. Inclusion or exclusion of outliers did not change results, and therefore outliers were retained for analyses. No corrections were needed for heterogeneity of variance (112).

Across the intervention group programs, 10 of the 15 participants attended all four group sessions (67%), and 5 participants attended three group sessions (33%). Five waitlist participants attended the intervention offered after completing their third assessment (39%).

### Participant characteristics

Participant demographic and medical data at Time 1 are shown in Table 2. There were no statistically significant differences among the groups in any of the demographic or medical variables. The two cancer groups also did not differ significantly from each other at Time 1 on any of the cognitive or psychosocial measures.

**Table 2:** **Participant characteristics**.

Variable	Intervention (*n* = 16)		Waitlist (*n* = 13)		Community (*n* = 16)	
	*M* (SD)	Range	*M* (SD)	Range	*M* (SD)	Range
Age (years)	50.4 (8.8)	37–65	51.8 (9.4)	40–72	52.9 (17.0)	27–77
Education (years)	15.8 (4.0)	11–26	13.8 (3.5)	9–20	13.9 (3.8)	10–20
Time since cancer diagnosis (months)	46.1 (22.8)	15–87	69.2 (56.5)	14–189	–	–
Time since finished cancer treatment (months)	37.1 (24.6)	6–84	46.5 (46.1)	6–137	–	–

	**%**		**%**		**%**	

Living with partner	68.8		84.6		75.0	
Female	93.8		100.0		93.8	
Born in Australia	62.5		76.9		56.3	
Neurological history	0.0		6.3		0.0	
Cancer type
Breast	75.0		76.9		–	
Hematological	6.3		15.4		–	
Colorectal	6.3		7.7		–	
Prostate	6.3		–		–	
Ovarian	6.3		–		–	
Previous treatment
Chemotherapy	81.3		100.0		–	
Radiotherapy	81.3		84.6		–	
Surgery	87.5		84.6		–	
Other	81.3		69.2		–	
Hormone treatment (current)	68.8		69.2		–	

### Cognitive results

#### Objective cognitive function

Descriptive statistics and RCT effect sizes for objective and subjective cognitive measures are shown in Table 3. At Time 1, only one cognitive measure, the RBANS Visuospatial/Constructional measure, showed a significant difference among the groups, *F* (2, 41) = 4.45, *p* = 0.018. This effect occurred because the intervention group scored significantly worse than the community group, *p* = 0.031, and the waitlist group showed a trend toward worse performance than the community group, *p* = 0.063.

**Table 3:** **Effect sizes, means (and standard deviations) for cognitive measures**.

Measure	*d*_Int−Wait_		Time 1			Time 2			Time 3	
	T2/T3		Intervention (*n* = 15)	Waitlist (*n* = 13)	Community (*n* = 16)	Intervention (*n* = 15)	Waitlist (*n* = 13)	Community (*n* = 16)	Intervention (*n* = 15)	Waitlist (*n* = 12)
Neuropsychological battery (RBANS)
Total scale	0.19∕	0.32	97.2 (10.2)	94.5 (13.2)	98.3 (12.8)	106.4 (9.6)***	101.5 (13.7)**	94.6 (13.9)	110.5 (10.4)***	104.0 (10.0)***
Immediate memory	0.40∕	−0.04	97.7 (11.6)	97.2 (14.4)	100.5 (16.6)	104.3 (14.9)	98.4 (17.7)	99.3 (18.5)	112.3 (17.1)**	112.2 (10.2)**
Visuospatial/constructional	0.11∕	0.79	88.3 (12.8)	89.3 (14.3)	101.9 (15.1)	97.1 (14.1)*	96.5 (17.1)^†^	91.4 (18.3)**	98.5 (12.3)	88.5 (12.6)
Language	−0.16∕	0.13	102.5 (15.1)	97.3 (7.2)	94.8 (14.0)	108.6 (6.9)^†^	105.5 (11.2)*	102.3 (11.6)*	107.2 (10.1)	100.4 (8.1)
Attention/concentration	−0.25∕	−0.18	106.6 (13.7)	101.0 (17.9)	98.9 (15.3)	109.9 (10.9)	108.3 (13.1)*	101.1 (17.1)	112.8 (8.9)^†^	110.2 (16.3)**
Delayed memory	0.49∕	0.06	96.9 (8.8)	96.3 (17.3)	98.9 (11.1)	104.8 (12.3)**	97.5 (13.0)	101.5 (14.8)	106.5 (7.8)***	105.1 (12.9)**
Trail making test (TMT)
TMT A	0.40∕	**0.58**	32.9 (7.4)	34.7 (12.9)	30.5 (8.8)	24.1 (5.2)**	30.2 (10.4)	26.5 (7.7)	23.2 (5.1)***	31.2 (9.4)
TMT B	−0.06∕	0.14	54.9 (11.7)	63.9 (24.1)	67.5 (22.1)	52.3 (13.2)	60.2 (24.2)	63.3 (20.6)	48.7 (14.8)	60.3 (24.9)
Self report (FACT-Cog 3)
Perceived cognitive impairments	**0.67**/	0.31	33.7 (15.5)	34.4 (16.1)	75.4 (5.3)	45.6 (15.5)***	35.4 (17.0)	73.4 (8.5)	50.2 (15.2)***	46.0 (17.0)*
Comments from others	0.26∕	−0.20	12.7 (1.8)	12.0 (3.8)	15.4 (0.9)	14.5 (1.9)**	13.1 (2.2)^†^	15.8 (0.6)	13.9 (12.1)	13.8 (3.4)^†^
Perceived cognitive ability	0.65/	0.68	13.5 (6.1)	16.2 (6.4)	32.1 (3.9)	17.5 (6.8)**	16.3 (6.6)	30.8 (6.8)	18.6 (8.1)*	16.9 (8.7)
Impact on QoL	0.13∕	0.22	8.8 (3.6)	9.3 (3.2)	15.6 (0.9)	10.3 (4.8)*	10.3 (3.3)	15.2 (1.8)	12.3 (4.6)***	12.0 (2.3)**
Self report (BAPM)
Instrumental activities	−0.15∕	0.56	19.5 (4.5)	22.2 (8.5)		18.8 (7.2)	20.5 (9.6)		17.9 (7.9)	16.8 (6.4)^†^
Basic activities	0.13∕	0.06	11.9 (4.1)	12.2 (4.3)	–	12.4 (9.5)	13.3 (7.9)	–	10.7 (4.2)	10.8 (3.4)

^†^*p* < 0.10, *p < 0.05, **p < 0.01, ***p < 0.001 for within-group comparison to Time 1.

*Higher scores indicate better performance for RBANS and FACT-Cog 3. Lower scores indicate better performance for Trail Making and BAPM. *d*_Int−Wait_ = effect size for Intervention improvement corrected for Waitlist improvement. Effect sizes associated with statistically significant interactions for RCT participants (*p* < 0.05) are shown in bold text; effect sizes associated with interaction trends (*p* < 0.10) are underlined*.

For ANOVAs comparing the two cancer groups across three time points, there were no main effects of group on objective cognitive function. There was a main effect of time on all objective cognitive measures except for TMT B, indicating significant improvements over time. For TMT B, the time effect approached significance, *F* (2, 50) = 2.95, *p* = 0.062, $\eta_{p}^{2}=0.11$. There was a significant Group × Time interaction for TMT A, *F* (2, 50) = 5.88, *p* = 0.005, $\eta_{p}^{2}=0.19$. The interaction occurred because the two groups did not differ significantly in TMT A completion times at Time 1, *F* (1, 25) = 0.08, *p* = 0.785, $\eta_{p}^{2}=0.00$ whereas the intervention group was significantly faster than the control group at both Time 2, *F* (1, 25) = 4.77, *p* = 0.038, $\eta_{p}^{2}=0.16$, and Time 3, *F* (1, 25) = 7.98, *p* = 0.009, $\eta_{p}^{2}=0.24$. No other objective cognitive measure showed a Group × Time interaction when analyzed for the two cancer groups only.

For ANOVAs that included cancer and community groups from Time 1 to Time 2, there were no main effects of group on objective cognitive function. There was a main effect of time on several variables, in each case indicating significant improvements over time. These time effects occurred for the RBANS Total score, *F* (1, 41) = 14.55, *p* < 0.001, $\eta_{p}^{2}=0.26$, RBANS Language Index, *F* (1, 41) = 10.97, *p* = 0.002, $\eta_{p}^{2}=0.21$, and TMT A, *F* (1, 41) = 11.50, *p* = 0.002, $\eta_{p}^{2}=0.22$. RBANS Total also showed a Group × Time interaction, *F* (2, 41) = 5.34, *p* = 0.009, $\eta_{p}^{2}=0.21$. The interaction occurred because there were significant improvements on RBANS Total from Time 1 to Time 2 for intervention, *F* (1, 41) = 16.10, *p* < 0.001, $\eta_{p}^{2}=0.28$, and waitlist, *F* (1, 41) = 7.90, *p* = 0.008, $\eta_{p}^{2}=0.16$, but not community participants, *F* (1, 41) = 0.11, *p* = 0.737, $\eta_{p}^{2}=0.00$. A Group × Time interaction for the Visuospatial/Constructional index, *F* (2, 41) = 9.88, *p* < 0.001, $\eta_{p}^{2}=0.33$ occurred because the cancer groups scored significantly worse than the community group at Time 1 (as noted above), but there was no difference among the groups at Time 2, *F* (2, 41) = 0.54, *p* = 0.585, $\eta_{p}^{2}=0.03$. Delayed Memory showed a trend toward a Group × Time interaction, *F* (2, 41) = 3.02, *p* = 0.060, $\eta_{p}^{2}=0.13$. Simple effects testing showed that Delayed Memory improved significantly from Time 1 to Time 2 for the intervention group, *F* (1, 41) = 9.01, *p* = 0.005, $\eta_{p}^{2}=0.18$, but not for waitlist, *p* = 0.687, or community participants, *p* = 0.771. No other objective cognitive measure showed a Group × Time interaction when analyzed across all three participant groups.

Clinical significance and reliable change scores were calculated for changes from Time 1 to Time 2 and Time 1 to Time 3, respectively, in order to more fully understand changes over time for individuals in each of the groups. Fisher’s Exact Test showed significant differences among the groups in the frequency of reliable change categories for the RBANS visuospatial/constructional scale at Time 2 (*p* = 0.030) and TMT A at Time 3 (*p* = 0.002). The difference in category frequencies for Visuospatial/Constructional scores at Time 3 approached statistical significance (*p* = 0.072). Visuospatial/Constructional scores showed reliable improvement or recovery at Time 2 for 27, 13, and 6% of intervention, waitlist, and community participants, respectively, with an unexpected reliable deterioration for 31% of community participants compared with 0% of cancer survivor participants. By Time 3, 47% of intervention and 8% of waitlist participants were classified as recovered on the Visuospatial/Constructional score. For TMT A, 0% of intervention participants, 13% of waitlist participants and 6% of community participants showed reliable improvement or recovery at Time 2. By Time 3, 46% of intervention and 0% of waitlist participants showed recovery on TMT A.

#### Subjective cognitive function

Descriptive statistics and RCT effect sizes are shown in Table 3. Both cancer groups reported significantly worse subjective cognitive function than the community group at Time 1, on all four FACT-Cog 3 subscales (all *p*s < 0.001).

For ANOVAs comparing the two cancer groups across three time points, there were no main effects of group on subjective cognitive function. There was a main effect of time on all FACT-Cog 3 measures, indicating significant improvements over time in these subjective cognitive functions. For BAPM-instrumental activities, a time effect approached significance, *F* (2, 50) = 3.14, *p* = 0.083, $\eta_{p}^{2}=0.10$. There was a trend toward a Group × Time interaction for FACT-Cog perceived cognitive impairments, *F* (2, 50) = 3.14, *p* = 0.052, $\eta_{p}^{2}=0.11$. This trend was associated with significantly reduced perceived impairments for the intervention group at Time 2, *p* = 0.002, and Time 3, *p* < 0.001, in comparison with the waitlist group showing no change at Time 2, *p* = 0.999, but significant improvement at Time 3, *p* = 0.013. There were no statistically significant effects for the measures of subjective prospective memory (BAPM), which were assessed in cancer survivor groups only.

For ANOVAs that included cancer and community groups from Time 1 to Time 2, there were main effects of group on all FACT-Cog 3 subscales, indicating worse subjective cognitive function reported by the cancer survivor groups than the community comparison group. There were main effects of time for perceived cognitive impairments, *p* = 0.025, and comments from others, *p* = 0.001, and trends toward time effects for perceived cognitive ability, *p* = 0.070, and impact on QoL, *p* = 0.083. In each case, the time effects indicated better subjective cognitive function over time. There was a Group × Time interaction for Perceived Cognitive Impairments, *F* (2, 40) = 7.72, *p* = 0.001, $\eta_{p}^{2}=0.28$. The interaction occurred because the intervention group reported significant improvement on Perceived Cognitive Impairment at Time 2, *F* (1, 40) = 20.39, *p* < 0.001, $\eta_{p}^{2}=0.34$, whereas the other groups did not change significantly (waitlist *p* = 0.736, community *p* = 0.448). Impact on QoL showed a trend toward a Group × Time interaction, *F* (2, 40) = 2.47, *p* = 0.097, $\eta_{p}^{2}=0.11$. Simple effects testing showed that Impact on QoL improved significantly from Time 1 to Time 2 for the intervention group, *F* (1, 40) = 5.25, *p* = 0.027, $\eta_{p}^{2}=0.12$, but not for waitlist, *p* = 0.170, or community participants, *p* = 0.484. No other subjective cognitive measure showed a Group × Time interaction when analyzed across all three participant groups.

Changes for individuals according to clinical significance and reliable change criteria showed a significant difference among groups in categories of change from Time 1 to Time 2 in perceived cognitive impairments, *p* = 0.002. There was clinically significant improvement or recovery on perceived cognitive impairments by Time 2 for 60, 17, and 0% of intervention, waitlist, and community participants respectively. By Time 3, perceived cognitive impairments showed clinically significant improvement or recovery for 73% of intervention and 50% of waitlist participants. A difference among groups also occurred in change categories for comments from others for Time 1 to Time 2, *p* = 0.047. There was clinically significant improvement or recovery on this measure for 27% of intervention, 18% of waitlist, and 0% of community participants at Time 2. At Time 3, clinically significant improvement or recovery in comments from others was seen in 13% of intervention and 27% of waitlist participants, respectively. There were trends toward differences in change categories for the BAPM-instrumental measure at Time 2, *p* = 0.075, and Time 3, *p* = 0.075. These effects for the instrumental measure were associated with reliable change in waitlist participants only and were the same at both time points: one waitlist participant recovered (8 cf. 0% for intervention group) and two other waitlist participants who were classified as “false positive” (17 cf. 0% for intervention group).

### Psychosocial results

Descriptive statistics and RCT effect sizes are shown in Table 4. There were significant group effects at Time 1 for distress (*p* < 0.001), QoL (*p* < 0.001 to *p* = 0.021), and fatigue (*p* = 0.005). For distress, emotional function, cognitive function, and social function, both cancer groups reported significantly worse function at Time 1 than the community group. For physical function, role function, global QoL, and fatigue, the intervention group reported significantly worse Time 1 function than the community group and the waitlist group did not differ significantly from the other two groups.

**Table 4:** **Effect sizes, means (and standard deviations) for psychosocial measures**.

Measure	*d*_Int-Wait_	Time 1			Time 2			Time 3	
	T2/T3	Intervention (*n* = 15)	Waitlist (*n* = 12)	Community (*n* = 16)	Intervention (*n* = 15)	Waitlist (*n* = 12)	Community (*n* = 16)	Intervention (*n* = 15)	Waitlist (*n* = 12)
QoL (QLQ–C30)
Physical	−0.06/0.13	80.9 (24.9)	81.1 (18.4)	97.9 (4.0)	84.4 (17.6)	86.1 (10.4)*	98.3 (14.8)	88.9 (14.2)	86.1 (16.4)
Role	0.40/0.28	72.2 (27.2)	79.2 (23.7)	93.8 (14.8)	85.6 (29.5)*	81.9 (19.4)	96.9 (9.1)	81.1 (32.0)	80.6 (15.6)
Emotional	0.00/−0.13	56.7 (28.2)	59.7 (15.8)	87.5 (12.9)	69.4 (27.6)*	69.4 (19.2)	88.4 (12.1)	70.6 (29.7)*	73.6 (13.2)*
Cognitive	0.37/0.17	55.6 (18.6)	59.7 (19.4)	96.9 (6.7)	65.6 (21.3)*	62.5 (19.0)	96.9 (6.7)	70.0 (28.9)^†^	70.8 (14.4)
Social	0.63/0.64	53.3 (24.6)	68.1 (26.1)	99.0 (4.2)	71.1 (28.5)**	69.4 (24.5)	96.9 (9.1)	81.1 (28.1)***	79.2 (17.6)
Global QoL	0.32/0.39	61.7 (23.1)	68.1 (15.6)	82.8 (15.1)	71.7 (22.0)*	71.5 (14.8)	85.4 (13.8)	73.9 (20.4)*	72.2 (13.9)
Fatigue	0.08/0.53	51.1 (27.1)	45.8 (19.0)	22.9 (20.1)	42.2 (23.5)^†^	38.9 (17.9)	14.6 (14.8)^†^	42.2 (28.1)	50.0 (26.6)
Distress (K10)	0.38/0.10	21.2 (7.0)	20.8 (5.2)	12.6 (2.6)	17.9 (7.4)**	19.8 (4.5)	12.2 (2.1)	18.4 (10.4)	19.3 (3.1)
Benefit finding	0.48/0.06	58.1 (13.8)	65.6 (12.2)	–	64.7 (9.4)^†^	64.7 (10.0)	–	60.8 (16.7)	67.4 (11.0)
CSE	0.13/**0.18**	39.1 (12.3)	43.6 (8.5)	–	42.3 (12.6)	43.8 (8.2)	–	47.0 (9.7)***	46.0 (5.7)
Illness perceptions (BIPQ)	0.36/0.45	45.9 (10.3)	43.1 (11.4)	49.3 (12.4)	37.4 (13.2)*	38.5 (9.5)	40.0 (9.0)**	35.6 (18.0)***	37.7 (11.3)

^†^*p* < 0.10, *p < 0.05, **p < 0.01, ***p < 0.001 for within-group comparison to Time 1.

*Higher scores indicate better QoL for all QLQ–C30 measures except fatigue; more benefit finding; and better CSE. Lower scores indicate less QLQ–C30 fatigue, less distress on K10 and more functional illness perceptions. *d*_Int−Wait_ = effect size for Intervention improvement corrected for Waitlist improvement. Effect sizes associated with statistically significant interactions for RCT participants (*p* < 0.05) are shown in bold text; effect sizes associated with interaction trends (*p* < 0.10) are underlined*.

For ANOVAs comparing the two cancer groups across three time points, there were no main effects of group on psychosocial measures. Seven of the 11 psychosocial measures showed time main effects, indicating statistically significant improvements over time in physical function, emotional function, subjective cognitive function, social function, global QoL, CSE, and illness perceptions. The time main effect approached significance for fatigue, *F* (2, 50) = 3.16, *p* = 0.051, $\eta_{p}^{2}=0.11$, and K10 distress, *F* (2, 46) = 2.90, *p* = 0.065, $\eta_{p}^{2}=0.11$. There was no effect of time for either role function or benefit finding. There was a trend toward a Group ×Time interaction for social function, *F* (2, 50) = 2.52, *p* = 0.091, $\eta_{p}^{2}=0.09$. This trend was associated with statistically significant improvement in social function over time for the intervention group, *F* (2, 24) = 10.87, *p* < 0.001, $\eta_{p}^{2}=0.48$, but not the waitlist group, *F* (2, 24) = 1.92, *p* = 0.168, $\eta_{p}^{2}=0.14$. There were no other interactions for the two cancer survivor groups on the psychosocial measures.

For ANOVAs that included cancer and community groups from Time 1 to Time 2, there were main effects of group on all psychosocial measures except for illness perceptions. These main effects indicated worse QoL and more distress for cancer survivors than for the community group. There were also main effects of time, indicating significant improvement on physical function, role function, emotional function, global QoL, fatigue, distress, and illness perceptions. Social function showed a trend toward a time effect, *F* (1, 40) = 3.69, *p* = 0.062, $\eta_{p}^{2}=0.08$, leaving subjective cognitive function as the only psychosocial measure that did not have a time main effect across the three groups. There was a Group × Time interaction for social function, *F* (2, 40) = 4.54, *p* = 0.017, $\eta_{p}^{2}=0.19$. The interaction occurred because the intervention group improved significantly in social function, *F* (1, 40) = 12.74, *p* = 0.001, $\eta_{p}^{2}=0.24$, but there was no change for the waitlist, *F* (1, 40) = 0.06, *p* = 0.804, $\eta_{p}^{2}=0.00$, or community groups, *F* (1, 40) = 0.19, *p* = 0.668, $\eta_{p}^{2}=0.01$.

There was a trend toward a Group × Time interaction for distress, *F* (2, 39) = 2.46, *p* = 0.099, $\eta_{p}^{2}=0.11$. This trend was associated with a significant reduction in distress from Time 1 to Time 2 for the intervention group, *F* (1, 39) = 10.69, *p* = 0.002, $\eta_{p}^{2}=0.22$, but no change for the waitlist, *F* (1, 39) = 0.68, *p* = 0.414, $\eta_{p}^{2}=0.02$, or community groups, *F* (1, 39) = 0.15, *p* = 0.698, $\eta_{p}^{2}=0.00$. There was no indication of interaction effects on other psychosocial measures when analyzed across all three participant groups.

Changes for individuals according to clinical significance and reliable change criteria showed a trend toward a difference among groups in categories of change from Time 1 to Time 3 in K10 distress, *p* = 0.059. From Time 1 to Time 2, one waitlist participant showed reliable improvement in distress (8%) compared to 0% of intervention and community participants. From Time 1 to Time 3, there was clinically reliable recovery (five participants) or improvement (two participants) in distress for intervention participants (50% of intervention participants), compared to zero waitlist participants recovered and three improved (25%). However, there was also clinically reliable worsening of distress by Time 3 for two intervention participants (14%) and one waitlist participant (8%). No other psychosocial measures approached statistical significance for differences among participant groups in categories of change for individuals.

### Associations between cognitive measures and potential psychosocial mechanisms

In order to investigate the relationships between cognitive functioning and potential mechanisms of change, a number of correlations were calculated within the cancer survivor groups. Change scores from Time 1 to Time 2 were calculated for illness perceptions and CSE. These two sets of change scores were then investigated for correlations with group (intervention or waitlist) and with Time 1 to Time 3 change scores for objective and subjective cognitive measures (see Table 5).

**Table 5:** **Correlations between change in psychosocial predictors at Time 2 and change in cognitive measures at Time 3**.

Variable	CSE T2–T1	BIPQ T2–T1
	*r (p)*	*r (p)*
Group^a^	0.19 (0.349)	−0.29 (0.156)
Objective change scores Time 3 – Time 1
RBANS (Total)	0.22 (0.279)	−0.06 (0.777)
TMT A	0.17 (0.411)	0.24 (0.248)
TMT B	−0.14 (0.480)	−0.10 (0.627)
Subjective change scores Time 3 – Time 1
FACT-Cog 3
Perceived cognitive impairments	0.30 (0.129)	−0.21 (0.301)
Comments from others	−0.11 (0.605)	−0.35 (0.086)
Perceived cognitive abilities	0.36 (0.068)	−0.36 (0.068)
Impact on QoL	0.45 (0.020)	−0.27 (0.188)
BAPM
IADL	−0.01 (0.981)	0.02 (0.937)
BADL	−0.06 (0.785)	0.20 (0.321)

*^a^Waitlist = 0, Intervention = 1*.

*Higher scores indicate better performance for RBANS, FACT-Cog 3, and CSE. Lower scores indicate better performance for TMT, BAPM, and Illness Perceptions*.

As Table 5 shows, there was a statistically significant correlation between improved CSE at Time 2 and improved FACT-Cog Impact on QoL between Time 1 and Time 3. There was a similar trend for CSE and FACT-Cog Perceived cognitive abilities, as well as trends toward correlations between improved illness perceptions and later improvements in FACT-Cog comments from others and perceived cognitive abilities. Correlations between the CSE and illness perceptions change scores with group assignment were not statistically significant, but were in the predicted directions (as were 17 of the 20 correlations in Table 5). The correlation between CSE change and illness perceptions change was not statistically significant, *r* (26) = –0.23, *p* = 0.269.

## Discussion

The current study used an RCT design to investigate the impact of a group intervention program targeting cancer-related cognitive dysfunction. Participants were cancer survivors who were randomly allocated to the intervention or waitlist, as well as a sample of people from the general public who participated as an additional non-randomized control group. The design incorporated evaluation of two potential psychological mechanisms of change, illness perceptions, and CSE.

Outcome measures that showed significantly larger effect sizes for intervention than waitlist participants were TMT A, perceived cognitive impairments from FACT-Cog 3, and CSE. Additional noteworthy trends indicating possible effects of the intervention occurred for perceived cognitive ability from FACT-Cog 3, social functioning, and fatigue. There were some indications of improved CSE and illness perceptions being associated with later improvements in subjective cognitive functioning.

### Objective cognitive function

There was partial support for Hypothesis 1, which predicted that participating in the intervention may lead to improvements in objective cognition. Intervention participants improved significantly more than waitlist participants in the time taken to complete TMT A. These results were consistent with the previous study assessing ReCog (75), and another study assessing the MAAT program (82), which also found significantly greater improvement for the intervention group on this measure as well as continued improvement from Time 2 to Time 3. These findings suggest ReCog to be beneficial for cognitive processes required in TMT part A, such as processing speed, visual scanning, and numeric sequencing. The current findings, in combination with results of previous research, also suggest that participants may continue to improve on some measures over a longer rather than shorter follow-up period after the intervention. This may be due to the practice-based nature of strategies included in ReCog and therefore further development of skills measured by these assessments over time. These findings are relevant in the context of literature indicating attention and concentration, processing speed, and executive functioning to be domains particularly vulnerable to problems following cancer treatment (113). For all other objective cognitive measures, there were main effects of time (or near-significant trends) for RCT participants, showing improvement over time for cancer survivors but no Group × Time interactions in ANOVA. Two previous RCTs have also found objective benefits for a cognitive rehabilitation intervention compared to waitlist to be limited to a single measure in the battery (86, 87).

Cancer survivors and community participants had equivalent objective cognitive performance at baseline except for the RBANS visuospatial/constructional index, which was significantly worse for cancer survivors. This finding was congruent with previous research indicating that visuospatial and verbal domains may be areas mostly affected by cancer and cancer treatments (12). Analyses of the community and cancer survivor data showed statistically significant interactions for RBANS total and visuospatial/constructional indices and a trend toward an interaction for delayed memory. These interactions were associated with bigger improvements for cancer survivors than for community participants, but with no difference in improvements between intervention and waitlist participants. The RBANS findings contrasted with a previous study of the same intervention, which found significant improvement on the RBANS total score for those who completed ReCog with a larger between-groups effect size when compared to the cancer comparison group than found in the current study (*d* = 1.00 vs. 0.19) (75). Research has suggested that effect sizes reported in pilot studies often differ from consequent RCT studies implementing the same interventions (114).

It is possible that both the intervention and waitlist groups showed improvement on RBANS measures due to practice effects or increased effort at re-testing. For the community group, although they may have also been motivated to participate due to awareness they were assisting with cancer survivorship research, they may not have been as motivated as the other two groups to do well or to improve their performance. Another interpretation of these findings could be that cancer survivors were less able to demonstrate their cognitive abilities at initial assessment and thus showed more benefit from repeat assessments than community participants, but the lack of objective cognitive baseline differences between cancer survivors and community participants on all but one measure makes this interpretation less likely. A further possibility is that participating in the assessments (with or without the intervention, and in the absence of feedback) helped give cancer survivors a sense that their cognitive performance was not as bad as they had initially thought, improving CSE, which may have helped them to improve their performance. This interpretation is consistent with improvements over time for both intervention and waitlist participants on subjective cognition and other psychosocial measures.

Looking at changes for individuals in relation to reliable change and clinical significance criteria, there were significant differences between groups in categories of change, with more intervention than waitlist participants showing “recovery” at Time 3 (TMT A 46 vs. 0% recovered; visuospatial/constructional index 47 vs. 8% recovered). These results were promising and indicated greater clinical improvement for participants who completed ReCog. Unexpectedly, 31% of community participants showed reliable “deterioration” on the visuospatial/constructional index at Time 2 (as also reflected in a significant simple effect of time within the community group), which suggests that results on this measure should be interpreted with caution.

### Subjective cognitive function and other psychosocial measures

There was partial support for Hypothesis 2 regarding intervention effects on subjective cognition. Intervention participants improved significantly more than waitlist participants on FACT-Cog perceived cognitive impairments from Time 1 to 2, but both groups showed similar improvements from Time 1 to Time 3. By Time 3, 73% of intervention and 50% of waitlist participants showed clinically significant improvement or recovery on perceived cognitive impairments. Perceived cognitive ability showed a trend toward greater improvements for intervention than waitlist participants. Other FACT-Cog subscales tended to improve over time for both RCT groups. Again the improvements in the waitlist group may indicate improved awareness, and an improvement in perceptions, of cognitive functioning as a result of the testing sessions. The variable length of time since participants had completed cancer treatments (see Table 2) argues against improvement in the waitlist group being attributed solely to ongoing recovery. Measures of prospective memory showed no statistically significant effects, which differed from another intervention study in this area that demonstrated improvement on this construct (104), but a different measure was used in the current study.

Hypothesis 3, which proposed intervention effects on psychosocial variables, was partially supported in relation to the intervention group demonstrating trends toward greater improvement in social functioning and fatigue, as shown by effect size comparisons in Table 4. The social function trend replicated a finding from the previous study of ReCog, but in both studies the interpretation of this finding is tempered by the consideration that the intervention group tended to report poorer social function at baseline (75). The social support element of group therapy and inclusion of broad elements in ReCog including self-care issues such as fatigue management mean that these effects could plausibly be associated with the intervention.

In contrast to objective measures, all four subjective cognitive measures showed worse performance of the cancer survivors than community participants at baseline. Cancer survivors also reported significantly worse QoL, fatigue, and distress than community participants at baseline. These results were consistent with previous literature suggesting an impact of cancer and cancer-related treatments on these psychosocial areas of functioning, as well as the finding that discrepancies between cancer survivors and community participants are often greater on subjective than objective measures of cognitive function (9). Although many of the subjective cognitive and psychosocial measures improved over time for the cancer survivors in the study, they continued to report worse average functioning than demographically matched community participants on most self-report measures, as can be seen from descriptive statistics in Tables 3 and 4. It would be of interest to find out whether a longer intervention, individual treatment, or more focus on broader issues would result in a higher proportion of cancer survivors finishing an intervention or follow-up period in the same range on subjective cognition and psychosocial measures as people who have not experienced cancer.

### Potential mechanisms

There was partial support for Hypotheses 4 (regarding intervention effects on illness perceptions and CSE) and 5 (regarding changes in illness perceptions and CSE predicting other intervention effects). CSE showed a significantly greater effect size for the intervention than waitlist group, and simple effects of time within group showed that both illness perceptions and CSE improved significantly in the intervention but not the waitlist group. If an intervention such as ReCog can deliver benefits by activating or strengthening participants’ beliefs that they are capable of improving cognitive performance by means they can control (such as use of skills practice or compensatory strategies), this would be expected to be reflected in improvements on variables such as illness perceptions and CSE and there was some evidence for this in the findings. However, neither variable could be directly compared with community data at baseline, because the measures were linked to perceptions of existing cognitive problems. To provide some basis of comparison, community participants reported illness perceptions in relation to a hypothetical scenario: what they thought it would be like if they experienced cognitive difficulties. Cancer survivors, in contrast, reported illness perceptions in relation to the cognitive problems that they actually experienced (as an inclusion criterion for the study). The illness perceptions that cancer survivors reported were no worse than what community participants imagined these problems would be like.

The occurrence of several statistically significant or near-significant correlations between earlier change scores for CSE or illness perceptions and later change scores for subjective cognitive FACT-Cog-3 subscales gave some support for these variables as potential mediators of intervention effect. The importance of mindset to self-reported cognitive problems in cancer survivors has previously been demonstrated via effects such as priming (115). However, these correlations could also indicate the reverse direction of causality or a third, common cause of change in both sets of variables. Larger studies are needed for more rigorous investigation of potential mediators of effect.

### Strengths and limitations

The study had a number of strengths. Groups were well matched and included a non-cancer group for further understanding of change over time as well as providing data for computing reliable change and clinical significance. Retention of participants was high across all groups. Assessors were unaware of group allocation for RCT participants serving to minimize observer bias. The study used a previously reported, manualized intervention and included assessment of potential mechanisms of intervention effect. The study was also the first to include an adapted version of the Traumatic Brain Injury Self-Efficacy Scale to assess CSE in cancer survivors.

A limitation of the current study is that the community group did not complete all of the measures administered to the treatment and waitlist group, and community participants did not complete a third assessment point. These issues occurred due to the community data being collected prior to the final selection of RCT measures, and this has limited comparison of certain measures and follow-up effects to the cancer groups only. The sample size in the current study was also relatively small, which is similar to most previous studies in this area, and impacts the power of analyses to detect smaller effects that may nevertheless be of clinical significance. The risk of Type 1 error was adjusted within analyses of each variable (e.g., use of simple effects analysis with a pooled error term and Bonferroni correction) but not between variables. For studies of this kind with larger sample sizes, it may also be beneficial to consider stratification by types of cancer and/or treatment during the randomization stage since these factors may influence the results. Results for the visuospatial/constructional index should be interpreted with caution, because the community participants unexpectedly performed significantly worse on this index at retest, suggesting variability in performance on this index may have been affected by factors other than practice and intervention effects. One possibility is that community participants may have felt less motivated to perform at their best at retest, but an RBANS “effort index” comprised of tests independent of the visuospatial/constructional scale (116) did not detect diminished effort among community participants with significantly worse visuospatial/constructional performance at retest. The need for subjective scoring of one of the subtests in the measure also does not fully account for the decrease, because it was also seen in performance on an objectively scored subtest in the same index. Another consideration in future studies could be to assess the use of hormonal replacement therapy in community participants, since this may impact outcomes. A final limitation of the current study was the use of a waitlist rather than an active control group, which increases the possibility of other factors contributing to the results and limits the ability to attribute changes to the intervention. The use of an active control group, and providing participants who are not immediately engaged in an intervention with some form of activity in the meantime, may control for issues associated with potential expectation of benefit, time of testing, and attention. This methodological improvement would provide a stronger research design and allow interpretation of effects as being more clearly attributable to the intervention.

### Future directions

Overall, these findings have provided further insight into the utility of a psychological intervention for people experiencing cognitive dysfunction following treatment for cancer. There was evidence to support improvements in areas of subjective and objective cognitive function, as well as areas of psychosocial functioning, for those who completed the ReCog program. As the study was limited by small sample size, it is difficult to generalize the results to the broader population of people who experience cancer-related cognitive dysfunction. However, the improvements observed in the intervention group warrant further investigation of interventions such as ReCog. Formal assessment of economic costs and benefits would be helpful; the main costs associated with the intervention are staff time and other resources required are few, although the use of more costly or extensive assessments would increase the resource requirements.

All intervention studies reported in this paper have found some degree of improvement in objective and/or subjective cognitive performance for post-treatment cancer survivors who are retested over time. However, improvements also frequently occur in participants who are waitlisted or who take part in interventions that are not specifically targeted toward cognitive rehabilitation. It will be important for future research to more clearly define active ingredients of interventions and to consider alternative approaches of assisting cancer survivors with this issue. Careful selection of comparison conditions and studies with larger number of participants will be important in this endeavor.

## Conflict of Interest Statement

The authors declare that the research was conducted in the absence of any commercial or financial relationships that could be construed as a potential conflict of interest.
